# Facilitators and Barriers for Young Medical Doctors Writing Their First Manuscript for Publication

**DOI:** 10.3390/ijerph18168571

**Published:** 2021-08-13

**Authors:** Rie Raffing, Thor Bern Jensen, Sanne Larsen, Lars Konge, Christine Møller, Hanne Tønnesen

**Affiliations:** 1 WHO Collaborating Centre, Clinical Health Promotion Centre, The Parker Institute, Bispebjerg & Frederiksberg Hospital, University of Copenhagen, DK-2400 Copenhagen, Denmark; tbje@sst.dk (T.B.J.); hanne.tonnesen@regionh.dk (H.T.); 2 WHO Collaborating Centre, Clinical Health Promotion Centre, Health Sciences, Lund University, SE-20502 Malmo, Sweden; 3 Communication Department, Danish Health Authority, DK-2300 Copenhagen, Denmark; 4 Centre for Internationalisation and Parallel Language Use, Faculty of Humanities, University of Copenhagen, DK-2300 Copenhagen, Denmark; sannela@hum.ku.dk; 5 Centre for HR and Education, Copenhagen Academy for Medical Education and Simulation (CAMES), The Capital Region of Denmark, DK-2100 Copenhagen, Denmark; lars.konge@regionh.dk; 6 Medical Manuscripts, DK-3540 Lynge, Denmark; medicalmanuscripts@gmail.com

**Keywords:** medical doctors, scientific article, facilitators, barriers, medical education, manuscript writing

## Abstract

Although scientific publication is often mandatory in medical professions, writing the first research article for publication is challenging, especially as medical curricula have only a minor focus on scientific writing. The aim was therefore to identify facilitators and barriers experienced by medical doctors writing their first scientific article for publication. An explorative inductive approach made use of semi-structured interviews for collecting data until saturation. Data were analyzed with systematic text condensation. Several barriers were identified: (a) writing in general; (b) writing in English; (c) dealing with content, structure, and presentation; and (d) navigating in the author group. Good supervision in the initial writing phase was a facilitating factor. Medical doctors requested a course in which they could work on their own articles and give feedback to fellow students. They valued skilled lecturers and individual supervision, and they wanted to learn about author instructions, how to present text correctly, and how to sell their core message. Their goal was to create a useful end product and to obtain European Credit Transfer System (ECTS) points. The facilitators and barriers that medical doctors experience when writing their first scientific article for publication and their course requests should be reflected in the learning objectives and content of future courses.

## 1. Introduction

Medical teaching curricula are being developed to comply with the latest evidence and meet the current needs of patients as well as of society in general. Throughout the world, countries collaborate on structuring their medical curricula around a framework of abilities known as CanMEDs, which recognize the variety of roles a medical doctor is expected to hold [1]. One of these roles is that of a scholar: a medical doctor who is engaged in continuous learning and teaching, evaluating evidence, and contributing to scholarship [2]. One important activity of a scholar is producing evidence and having it disseminated via publication in scientific peer-reviewed journals, which are usually written in English.

Publication of results is a general requirement for all researchers, but writing the first scientific article for publication is still challenging for novices across all disciplines, including medical doctors [3]. An important challenge is the barrier to initiating the writing process itself. With little experience in scientific writing, over 50% of medical students find initiating the writing process of their 10-page Bachelor of Arts (BA) assignment problematic [4]. This challenge continues into the postgraduate period. PhD supervisors report that helping medical PhD students write their first article is more difficult than helping students from other disciplines [3]. The language barrier presents a general challenge for those who are not fluent in English [5,6]. One solution is to use language editing services. This solution can be costly for a researcher with limited resources and might therefore not always be entertained, nor might it be enough. Another solution is to provide courses in written English specifically for health science researchers [7]. Collectively, these studies suggest that the acquisition of the necessary skills and knowledge to approach the writing process is a complex matter in need of further research. The purpose of this study is therefore to explore what the medical doctors themselves see as barriers to initiating the writing process.

Barriers and facilitators affecting the writing process of medical doctors, including PhD students with a medical background, have been sparsely investigated. Other researchers have looked at facilitators and barriers of medical doctors when writing a full manuscript from an abstract, but as they have included medical doctors doing research outside an official PhD program, the main barrier they have identified is lack of time [8,9]. This study includes PhD students with a medical background and focuses on the difficulties with getting started on the actual writing process.

It is highly relevant to uncover the barriers and facilitators because tailored support of medical doctors can only then be developed to improve their writing processes. This will be of significance not only for the students themselves, but also for supervisors, for journal editors receiving the manuscripts and for peer reviewers reading them. Shah has called for qualitative studies that can help novice researchers by identifying barriers to scientific writing [10]. Similarly, David Kern recommends including qualitative methods for problem identification and needs assessment of the targeted learners [11]. A good way to investigate the subject is to conduct semi-structured interviews as their strength is the use of predetermined, open-ended questions that permit the interviewer to deviate and pursue paths important for the interviewee [12]. The aim of this study was therefore to identify facilitators and barriers experienced by medical doctors writing their first scientific manuscript for publication.

## 2. Materials and Methods

### 2.1. Methodology

This study was part of The Language Strategy: More Languages for More Students at the University of Copenhagen [13,14]. Participants were recruited for semi-structured interviews based on the survey from The Language Strategy [15], and by snowball sampling. Purposeful sampling was used to select the participants, i.e., they should share critical similarities that related to the aim of the study [12]. These critical similarities were defined as being a PhD student, having a medical background, and having relatively recent experience with writing their first scientific article. At the same time, obtaining rich data was attempted by selecting participants within a variety of medical research areas.

### 2.2. Data Collection

The semi-structured interview guide (see Appendix A) was developed specifically for this study by an expert in qualitative interview techniques and an information specialist. The guide was tested in a pilot interview. As this led to no significant changes to questions or techniques, the pilot interview was included in the data. The guide covered three overall areas: demographic questions; recollection of the experience of writing the first article for publication, preferably as the first author; and specific details about article writing. Interviews were carried out until saturation.

The interviews were conducted in the participants’ own setting, for example, at their workplace, in an online meeting room, and in private homes; except for on one occasion when the interview was conducted in the interviewer’s office.

### 2.3. Data Analysis

Interviews were recorded digitally and transcribed verbatim. Systematic text condensation was employed [16]. The transcriptions were coded using NVivo Qualitative Data Analysis Software, QSR International Pty Ltd., Melbourne, Australia. The coding gave rise to themes that guided the interpretation of the research findings in line with the research question. This aligns with an inductive approach to qualitative data analysis [17]. The analysis was performed by the first author, and the identified themes and interpretations were then discussed with a co-author to avoid bias.

### 2.4. Ethical Considerations

All participants were enrolled in the study after providing written informed consent. They were presented with the background and the purpose of the study both orally and in writing, and informed that they could withdraw from the study without any consequences. Potential participants were also given the opportunity to be informed of the results and to enroll in a tailor-made course based on these results. The data handling followed the guidelines for research from the Danish Data Protection Agency [18], now included in the General Data Protection Regulation (GDPR). This type of interview study is not subject to review by the Ethical Committee Board according to Danish legislation [19].

## 3. Results

The study included seven PhD students (six women and one man; age range: 30–34 years) (Table 1). In terms of their experience with writing for publication, they had published between one to seven scientific papers and at least one as a first author.

The interviews lasted from 28 to 69 min. Four main themes were identified in the interview data (Table 2): one theme was divided into four subthemes; another theme was divided into three subthemes. When the participants talked about the barriers to writing that they had experienced, it often sparked reflections on how to facilitate the writing process. To be included, a theme had to be mentioned by at least five of the seven participants.

### 3.1. Barriers

#### 3.1.1. Difficulties with Writing

Difficulties with writing were often presented. Beyond the PhD thesis itself, and owing to a curricular emphasis on reading textbooks, the participants reported sparse opportunities for learning the specific style and structure of scientific articles. Their related main concern was the potential for misinterpretation of their work. Participants also reported lacking an overview of the writing process, difficulties expressing themselves concisely and difficulties reflecting on the results as barriers to getting started writing:

“Yes, one has absolutely nothing to go on. Well, at SUND (Faculty of Medical and Health Sciences), you’ve only got a BA assignment and a Master assignment. That is kind of what you’ve got, there is not really anything in between, not at that time, anyway. And it is a very special way of writing and structure that you do not find elsewhere. One does not read many articles in the curricula in that way. In the curricula, much of it is textbooks and memorization, right? So, there is not much reading of articles, though I think there might have come more lately.”

(Participant 2)

#### 3.1.2. Difficulties Writing in English

Difficulties writing in English were mentioned in several different areas: British and American English, punctuation, vocabulary, grammar, conjunctions, and sentence structure. Lack of confidence, finding it difficult to be convincing through the medium of written English, and finally the challenge of going from layman’s English to the language of medicine were also mentioned.

“Well, I don’t think it was easy to write in English. Of course, you have some books, some texts in English at medical school, but you are not taught English as such and you do not have any assignments in English. Therefore, it has been many years since I have expressed myself in English, apart from what you do when on holiday. Moreover, I am certainly science-math oriented, so I’ve never found languages easy and I do not think they are particularly fun. So, it was a bit of a hurdle or something one should pull oneself together to do. I think it went well in the end; it was more a mental thing—there was a barrier, right?”

(Participant 7)

#### 3.1.3. Difficulties with Content, Structure, and Presentation

Difficulties with content, structure, and presentation were expressed as wide-ranging concerns. The participants were unsure about selecting content, including which results should be reported and for which target group. For example, they wondered what they should do if further significant findings in the data analyses were not relevant to the purpose of the article or the target group they were addressing. Other examples of uncertainty were the handling of incoherent results, and the order in which results should be presented. They felt they needed help to select and organize the content. Some found retrospective studies to be the most difficult to present.

“Of course, we had a guiding point, and we knew who to target, but once you’ve got data in your hands, a lot of new things are included. That also meant that I felt a bit alone with this and I remember that I met my supervisor 2–3 times where I presented some bar charts in Excel and said ‘Look, I’ve found this and that, and this is also interesting’. In the end, I needed somebody who kind of said, ’Now we decide that this is what we focus on‘ and my main supervisor did just that.”

(Participant 6)

All participants stressed that they found structuring the article challenging. They were familiar with IMRaD (Introduction, Methods, Results and Discussion) but found it difficult to decide what the different sections should contain. They found it particularly difficult when they did not know if there was a predefined structure, giving the case report as an example. They requested a form or list of what to include and in what order.

“I think the structure is difficult […] because I did not know all those things that I know today; that the first sentence of the discussion should summarize…well, just those kind of rules. I did not know where to put limitations and where to put strengths and where to put them in relation to each other. You know, there are so many rules that you are not familiar with until you’ve written many articles, I think. Even now I must look them up all the time.”

(Participant 4)

The participants mentioned that they were uncertain whether what they wrote was correct, especially when they did not have a supervisor at hand. They also found it challenging to report on qualitative studies in a quantitative format using the requisite number of words. Another difficulty was finding the balance between telling a good story and being biased: how should they deselect content to shape a core message while still being honest in reporting the results?

“What is the story here, and have you selected the good story? And then of course you must be honest in your results, in your bias, in your reservations and so on. But you can do that in many ways….”

(Participant 3)

#### 3.1.4. Difficulties Navigating in the Author Group

Difficulties navigating in the author group presented an overall challenge for inexperienced researchers who nevertheless reported that they had the most up-to-date knowledge in the author group that included supervisors. Disagreeing with a more experienced researcher was not easy. The participants admitted lack of confidence in themselves and their work. Apart from needing help with the actual wording of the text, they expressed a need to have someone else decide whether the content could be understood. Difficulties navigating within the author group also included lack of recognition and lack of input from co-authors, as well as disagreements regarding co-authorship. Managing the writing process, maintaining a good relationship with co-authors, and ensuring the optimal use of time were also mentioned as important.

“Well, one had…I had read more articles than them on this subject, newer articles, that is. So, I was just more updated. So, I observed how people wrote in the literature about things that the supervisors were not updated on. […] I just remember that the communication was a little heavy in the beginning where one did not really know how much authority one had, if one could really trust in oneself in it.”

(Participant 2)

### 3.2. Facilitators When Getting Started: Participants’ Tips

A second overall theme included the many tips the participants suggested as facilitators for getting the writing process started. These facilitators fell into three categories (Table 3).

### 3.3. Role of the Supervisor

This theme is an example of how an open-ended interview guide can lead to themes important for the interviewee that the interviewer had not foreseen. All the PhD students in the study emphasized that their supervisor was largely responsible for initiating a good and an as-easy-as-possible writing process. Elements of good supervision included swiftly answering questions so the student does not get stuck, agreeing on receiving imperfect drafts, finding focus/purpose in the data, giving concrete comments on the text, knowing the relevant literature, and—not least—being thorough.

“First of all, he had time, and he answered the emails. That is always an important thing. And he did not just read and say that it was fine. He was critical in his approach, and I think that is necessary when you are so early in your procedure. Because it is now [that] one needs to learn it. One can say that it is kind of an investment for them also. And then he wrote well himself.”

(Participant 5)

One participant mentioned that supervision of the writing process could benefit from being supported by a course that included writing tools.

### 3.4. Course Content

The participants showed great interest in a future course where the focus would be on getting started writing a scientific article. They all stated that such a course should include working on their own text and sharing feedback with others, either with a writing partner or in small groups, to deal with mutual problems (Table 4). They expected this would make the text easier to understand for readers without any pre-existing detailed knowledge.

“It should be possible to send your own things in to have them assessed, so one can get some feedback on one’s specific style. Also, examination of a research article with the different elements would be really good. Tips, introduction to abstract and what to think of there, yes, all parts really.”

(Participant 5)

## 4. Discussion

This study adds in-depth perspectives by identifying four main themes important for medical PhD students reflecting on their experience when initiating the writing process of their first article: (1) barriers, with the subthemes writing in general, writing in English specifically, selecting content, choosing structure and presenting material, and navigating in the author group; (2) facilitators, with the subthemes receiving advice on how to get started, writing in general, and writing in English specifically; (3) the role of the supervisor; and (4) course content requested by the participants.

### 4.1. Barriers

The participants pointed to several barriers when writing an article for publication. Some of these barriers are reflected in the international literature, whilst some are new findings. General difficulties for students with a medical background initiating the writing process itself have also been found in previous research [3,4,20]. Specifically, lack of writing experience was found in a previous study, suggesting that PhD students with a medical background did not receive sufficient training in writing as part of their curriculum [20,21], although possible solutions were not evaluated. In contrast to this study, other studies among hospital trainees, surgeons and researchers have identified further barriers to writing: mainly limited time, but also limited data, remote working, lack of logistical resources, cognitive burden, and perceiving plagiarism as a threat [8,9,10,20,22]. This difference might be explained by a difference in the participants’ circumstances, as the PhD students in this study had a structured program with a clear and approved timeline as part of their study plan.

All participants mentioned the English language as a barrier to writing. Another local study confirmed this and a global report on academic publishing in general also showed that non-native English speakers find their limited language proficiency to be a challenge in the publication process [5,6]. If English writing is not a major issue, it may be because the study participants are from English-speaking countries such as Australia, the USA, and Canada [9,10,21,23]. A way to overcome the language barrier would be to join courses on English scientific writing or to use language editing services.

Although challenges with content, structure, and presentation do not appear in the literature as major barriers to writing, one study reported that students found it difficult to distinguish between content and structure in article writing [10]. The reason these results are generally not found in the literature might be due to local conditions or because other studies have focused more on barriers of an institutional or relational character and less on the barriers experienced when actually putting pen to paper.

Challenges when navigating in the author group resembled what has been found in other studies [8,10,21]. A study about author groups and co-authorships especially focused on the problem of including underperforming contributors or excluding deserving contributors [24]. This problem was not pronounced in the data of this study, perhaps as the focus was on writing and not publishing an article. Lastly, the participants mentioned that they found it difficult to ensure up-to-date knowledge of senior members in an author group. This aspect has not yet been identified in other studies. 

### 4.2. Facilitators When Getting Started

The literature with tips and tricks on article writing is booming, but research on students’ self-reported suggestions to overcome barriers when getting started writing manuscripts is sparse [25]. Students from one study suggested using user-friendly documents and personal feedback [10]. Personal feedback from supervisors, co-authors, and networks in general was also suggested by the participants in this study. They did not mention user-friendly documents specifically, but they did stress the importance of a good outline and having a template for different sections of the manuscript. Overall, high-quality evaluation of the effect of the tips and suggestions is lacking.

Some studies have investigated specific methods for writing articles, such as writing groups or outlining and dictating [26,27]. Other studies have encouraged institutions to organize academic writing programs and workshops on manuscript writing, but have not reported on desired course content or a potential effect [9,23]. The need for specific training on how to write a good article has been recognized not only by students and supervisors but also by the editors-in-chief of major journals [28]. 

### 4.3. The Role of the Supervisor

Across the interviews, the participants stressed that a facilitating factor for the writing process was a supervisor who was easily accessible for early constructive feedback, which has also been reported in other studies [9,10]. It has been suggested that supervisors should be equipped with skills and guidelines to support students’ writing abilities and furthermore that students should expect supervisors to teach them how to write [23]. As mentioned, this part of supervision has not been evaluated so far and is in need of further investigation.

### 4.4. Course Content

There is an urgent call for better and more structured training in scientific writing earlier in the pre-graduate or post-graduate stage in line with the training of other medical skills such as patient communication, surgical techniques, and objective examinations. This could draw on the techniques of simulation training, which has proven effective in different settings and participating groups [29]. A new course with ECTS points building on the results of this study and the literature would have the following learning objectives: (1) to define the purpose of an article, (2) to select relevant content, (3) to structure content, and (4) to write in appropriate scientific English. The products would include (1) a qualified draft of the focus and purpose of the article, (2) a qualified draft of the content and structure, and (3) an identification of individual areas for improvement. The course would also provide the participants with useful writing tips, enabling them to meet the challenge of getting started with writing their first article. However, an evaluation process to increase knowledge in this field would be recommended. 

### 4.5. Bias and Limitations

This study employed purposeful sampling, including recruitment from a previous study and snowball sampling, which might have led to the overrepresentation of women. However, as about two-thirds of the PhD students within healthcare in Denmark are women (62.3% in 2018), this gender distribution seems acceptable [30].

To minimize the risk of confirmation bias, the interpretation of the findings was discussed by the two interviewers. Several of the themes identified have also been found in other studies, which adds to the credibility of the interpretation, and it is expected that these findings are relevant to all writing of a first article in a healthcare setting. The identification of needs not previously uncovered could be due to a confirmation bias or to local conditions. These findings would benefit from being confirmed or refuted in future studies.

Although it might be a limitation of the study that only seven participants were interviewed, participants were included until data saturation, which already occurred after seven interviews. All the themes identified in the analysis were mentioned by at least five of the seven participants and often by six or all seven, thereby strengthening the results. It was also a strength that all interviewees responded to the questions in the semi-structured interview guide and that they were given the time needed for an in-depth answer. The different lengths of the interviews depended mainly on how the interviewee responded to the questions and the extent to which the interviewer had to ask follow-up questions to cover the question adequately. Some interviewees responded clearly, briefly, and in short sentences, others responded with many words and gave elaborate explanations and examples.

### 4.6. Practice Implications and Perspectives

This study allowed PhD students with a medical background to communicate their needs respecting barriers and suggestions for facilitators when writing their first article. Furthermore, the study has also confirmed existing research in the area as well as contributed new findings.

The practical and experience-based result of barriers and facilitators for writing in English calls for intervention with practical implications for universities, supervisors, and students. Universities often benefit from their researchers publishing in international English-medium scientific journals as they are higher ranked than local language journals. Success in this endeavor, however, requires an increased focus on supporting PhD students’ writing skills in general through credit-bearing writing courses in addition to financial support for language editing. Supervisors should encourage their students to attend writing courses, and, based on joint decision-making, arrange for tailored supervision of their students’ first article already from the initial writing phase. Students should align expectations with their supervisors in order to get optimal support early in the writing process and join courses in medical writing to improve their writing skills. This is likely to result in an easier and less frustrating process of writing the first article and getting it published in a highly ranked journal and thus becoming a member of the international research society. While publishing in the local language is essential for reaching local stakeholders, a parallel strategy of publishing in English is necessary for reaching a broader audience. Based on the results, a tailor-made course has been developed and learning objectives and content have been targeted at this specific group. New research should repeat the interviews after piloting the requested new course. A customized training course in article writing accessible to PhD students can both improve and speed up the process of making research findings available to other researchers and to clinicians, patient groups, the general public, and policy-makers who are all eager to prevent, diagnose, treat and follow up on diseases in the best possible way. Ultimately, future patients can then resume their everyday lives in society.

## 5. Conclusions

Medical doctors experience facilitators and barriers when writing their first scientific article for publication. Good supervision was considered an especially important facilitating factor. Barriers included difficulties writing in general and writing in English, difficulties with content, structure and presentation, and finally difficulties navigating in the author group. The medical doctors provided considerable input regarding the content of a future course that would meet their needs. They requested a course where they could work on their own articles and get feedback from skilled lecturers and fellow students. They would like to learn more about author instructions, how to present text correctly, and how to sell their core message. Lastly, it was important for them to create a useful end product during the course and to obtain ECTS points. These requests should be reflected in the learning objectives and content of future courses.

## Figures and Tables

**Table 1:** Study participants interviewed on facilitators and barriers experienced when writing their first article for publication.

Participant	Gender	Age	Research Area	Number of Publications	Types of Authorships
1	Man	32	Pediatrics	1 publication	1 1st authorship
2	Woman	33	Pediatrics	7 publications	7 1st authorships
3	Woman	30	Cancer	1 publication	1 1st authorship
4	Woman	34	Diabetes	5 publications	2 1st & 3 co-authorships
5	Woman	30	Internal medicine	5 publications	2 1st & 3 co-authorships
6	Woman	31	Neurology	5 publications	5 1st authorships
7	Woman	33	Pediatrics	4 publications	4 1st authorships

**Table 2:** Themes and subthemes of interviews about writing the first article.

(1) Barriers to getting started
(a) Difficulties writing
(b) Difficulties writing in English
(c) Difficulties with content, structure and presentation
(d) Difficulties navigating in the author group
(2) Facilitators when getting started: participants’ tips
(a) How to get started
(b) Writing
(c) Writing in English
(3) Role of the supervisor
(4) Course content

**Table 3:** Facilitators when getting started: participants’ tips.

(a) How to get started Brainstorm! Just write something and get some early feedback. Have the courage to share imperfect drafts with the author group or your supervisor. Stick to the plan and first put references and points in every section. Begin with what you feel you can control. It might be the method section (“what we did”), then the results (“what we found”), then Introduction and Discussion. Know the message, content, points, and structure before you produce your text. Make a list of different sections to include and in what tense they should be written. Read 50 articles and pay attention to the language and structure. Make yourself familiar with the current structure of articles in the research area (read journal guidelines).
(b) Writing Identify the focus of the manuscript. Have a list of standard phrases that you can modify. Have a template for different sections of the manuscript. Seek specific advice on writing from your supervisor. Seek help from your network where this is easily accessible. Ask your author group for specific advice. Write your entire manuscript down on a piece of paper in four sentences: What is the purpose of the study? How did you do the study? What did you find? How can this be used in the future?
(c) Writing in English Read scientific articles in English to get to know the scholarly language and to identify good ways of saying and wording things. Join a course on scientific writing taught by a native English speaker. Receive concrete language suggestions from the author group and supervisor if they are good at written English. Accept the reality that you are an author who has English as a second language. It can even be a good thing that the language is uncomplicated. Have a good disposition from the beginning; this will give you time to look for words on the internet and in dictionaries. Keep your message simple. Consider scientific English to be a universal language for the research world. Get a native English speaker to revise the manuscript. If necessary, pay for language revision.

**Table 4:** Requested course content: Writing the first manuscript for publication.

Write and receive feedback on own texts Read and give feedback on the texts of others Work on concrete problems in course participants’ texts Revise the same passage repeatedly Work in small groups or with a writing partner Receive individual supervision Receive help from skilled and competent lecturers Consider author instructions for target journal Learn how to present information effectively (content, figures and tables) Learn how to sell the core message Produce a draft of the article Create an end product Obtain European Credit Transfer System (ECTS) points

## Data Availability

The data consist of interview transcriptions. As sharing these data could compromise individual privacy, they cannot be made publicly available.

## Appendix A

### Appendix A.1. Semi-Structured Interview Guide

Demographic questions

1. What is your name?
2. How old are you?
3. What is your educational background? Is this your only education?
4. How far are you in your PhD?
5. Which PhD program are you enrolled in?
6. What is the subject of your PhD?

### Appendix A.2. Experience with the First Article

7. Have you previously written a scientific article? Have you written more than one article? If so, how many?
8. What was your experience when writing your first scientific article?
9. What was easy (about the writing process)?
10. What was difficult (about the writing process)?
11. What kind of support did you receive?
12. Was that enough?
13. Was anything missing?
14. What was exceptionally good?
15. Do you feel comfortable writing in English?

### Appendix A.3. Specific Focus Areas

16. Looking at specific areas, did you find it easier or more difficult to focus on:
  (a) The aim of the article (specific and not the whole project)
  (b) The content (selection of material/brainstorming)
  (c) Structure (paragraphs, including paragraph headings)
  (d) Presentation (how it is written, including argumentation, tenses, etc.)

**Table A1:** Summing up: To what extent did you find the following difficult?

	Not at All	A Little	To some Extent	A Lot	Don’t Know
The aim					
The content					
The structure					
The presentation					

17. With respect to what you found difficult when writing your first scientific article, would an article writing course have made a difference for you?
18. Why?
19. What should such a course contain?
20. Would you choose a course in writing the first scientific article if it were offered?
21. Do you have any questions or is there any information you would like to add?
22. What will you take with you from this interview?
